# A rapid and cost-effective pipeline for digitization of museum specimens with 3D photogrammetry

**DOI:** 10.1371/journal.pone.0236417

**Published:** 2020-08-13

**Authors:** Joshua J. Medina, James M. Maley, Siddharth Sannapareddy, Noah N. Medina, Cyril M. Gilman, John E. McCormack

**Affiliations:** Moore Laboratory of Zoology, Occidental College, Los Angeles, CA, United States of America; University College London, UNITED KINGDOM

## Abstract

Natural history collections are yielding more information as digitization brings specimen data to researchers, connects specimens across museums, and as new technologies allow for more large-scale data collection. Therefore, a key goal in specimen digitization is developing methods that both increase access and allow for the highest yield of phenomic data. 3D digitization is increasingly popular because it has the potential to meet both aspects of that key goal. However, current methods overlook or do not prioritize some of the most sought-after phenotypic traits, those involving the external appearance of specimens, especially color. Here, we introduce an efficient and cost-effective pipeline for 3D photogrammetry to capture the external appearance of natural history specimens and other museum objects. 3D photogrammetry aligns and compares sets of dozens, hundreds, or even thousands of photos to create 3D models. The hardware set-up requires little physical space and around $3,000 in initial investment, while the software pipeline requires $1,400/year for proprietary software subscriptions (with open-source alternatives). The creation of each 3D model takes 1–2 hours/specimen and much of the software pipeline is automated with minimal supervision required, including the onerous step of mesh processing. We showcase the method by creating 3D models for most of the type specimens in the Moore Laboratory of Zoology bird collection and show that digital bill measurements are comparable to hand-taken measurements. Color data, while not included as part of this pipeline, is easily extractable from the models and one of the most promising areas of data collection. Future advances can adapt the method for ultraviolet reflectance capture and increased efficiency and model quality. Combined with genomic data, phenomic data from 3D models including photogrammetry will open new doors to understanding organismal evolution.

## Introduction

Natural history collections are experiencing a renaissance, as new analytical techniques are able to draw more information from each specimen [1–4], embodied in the concept of the Extended Specimen [5]. At the same time, digitization efforts connect these information-rich specimens across museum collections, allowing for the creation of large-scale biodiversity data sets [6–8]. Such large-scale biodiversity data have been used recently to show biological responses to climate change [4, 9] and to study broad-scale evolutionary patterns [10–12].

A key goal of efforts to connect large-scale biodiversity data across museums is the creation of tools that facilitate mass digitization [13, 14] while also providing the highest quality data, which can later be extracted by researchers [15] or through crowd-sourcing [11]. For the sheer amount of extractable data, it is no surprise that there has long been interest in 3D digitization in the world of natural history collections [16]. Currently, the most common 3D digitization techniques for natural history specimens are laser scanning and computerized tomography (CT) scanning [17, 18]. Laser scanning creates a 3D model through external tracking of the 3D position of a laser sight, whereas CT scanning uses penetrating waves that capture an image in 2D slices, which are then layered into a 3D model. While these methods provide a wealth of new data, neither captures one of the most sought-after features of specimens: full-color external phenotype.

Here, we outline a rapid and cost-effective method for obtaining 3D models of the external features of natural history specimens and other museum objects using digital photogrammetry. Digital photogrammetry (i.e., ‘Structure from Motion’) involves photographing an object from multiple angles, then using software that aligns common landmarks between the photographs to reconstruct a 3D model from sets of dozens, hundreds, or even thousands of photos [19]. First applied to landscapes and geological features [20, 21], 3D photogrammetry then moved to archaeology, paleontology, and cultural heritage sites [22–24], and eventually was improved to capture smaller and smaller objects [25, 26].

In natural history collections, insect specimens were the first to see applications of 3D photogrammetry, mostly directed toward type specimens [27–29]. Only very recently have a few lineage-specific applications emerged for the study of vertebrates like parrots [30], bats [31], and terrestrial mammals [32, 33]. Another recent development is Beastcam technology, a patented platform to elucidate live-animal motion and functional morphology using multi-camera 3D photogrammetry [34]. Despite this flurry of interest, to date, no large-scale 3D photogrammetry efforts have begun to mass-digitize specimens in natural history collections for the purpose of providing phenomic data for broad-scale evolutionary studies. In part, this is because the hardware and software described so far have been complicated, expensive, use-specific, or requiring considerable investment in staff time.

The 3D photogrammetry method we outlined below is broadly applicable and straightforward, relatively cheap, and largely automated in both its hardware and software, allowing for efficient digitization of biological specimens, even those with moderately complex structures. We show a simple use-case by comparing digital measurements from 3D models to those taken by hand from a series of bird holotypes (i.e., specimens representing the link between scientific names and phenotypes). We compare our pipeline to existing methods and discuss future directions.

## Methods

### Camera, hardware, and physical set-up

A detailed step-by-step guide to the entire procedure, which takes 1–2 hours per specimen including processing time (Table 1), can be found on Github at https://github.com/JMedina3D/MLZ-Museum-Photogrammetry-Protocol. For digital photography, we use a Sony a7rii camera for its large (42mp) sensor size and a 90mm macro lens for fine details. When capturing larger specimens, the macro lens is alternated with a 15mm wide-angle lens. We use a polarizing filter to remove excessive reflections and harsh shading in the final model.

**Table 1:** Average processing time for each component of the pipeline.

Stage	Average time
**i**. hardware setup	variable
**ii**. image capture	20 minutes
**iii**. image processing	20 minutes
**iv**. alignment/mesh reconstruction	Reality Capture: 20–30 minutes
**v**. manual mesh processing	5 minutes
**vi**. procedural mesh processing	5–10 minutes
**vii**. texture generation	10 minutes

For scanning large objects, it can be useful to combine multiple camera distances during image capture: medium-distance shots that frame the object’s form for geometric reconstruction, and close-up or zoomed-in shots for detail and texture. Since our objects were medium-to-small size, we use a high mega-pixel camera, which allows the same photoset to be used for both detail and structure. This cuts the number of photos in half, simplifies camera setup, and eases the process of automation. Additionally, a single camera position minimizes consistency errors in focal length or lighting.

**Fig 1:**
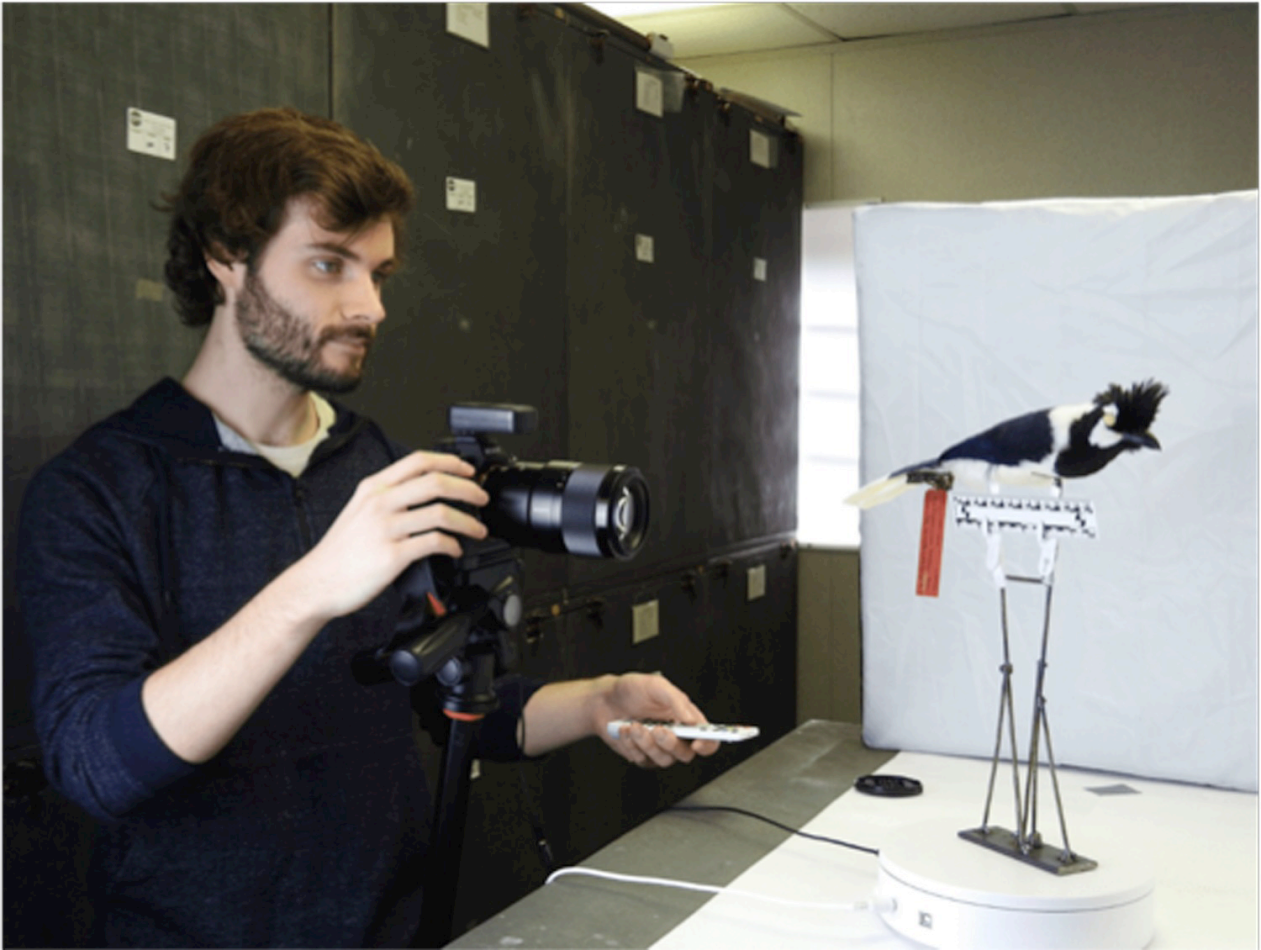
One of the authors with the physical set-up, showing the stand on top of a turntable with shutter integration to a camera on tripod. The ruler sits just below the specimen. Softbox light sources (one shown) are placed on both sides of the specimen. The specimen shown is the holotype of the Tufted Jay (*Cyanocorax dickeyi*), MLZ:Bird:12342.

The physical hardware includes a stand for holding the specimen, a turntable, and a matte backdrop. The stand is designed for small to medium-sized bird specimens, with four spokes that can be concealed within the feathers to allow for an unobstructed 360-degree view of the specimen (Fig 1). We place two lights (“softboxes”) on either side of the set-up to ensure even lighting. We use a motorized Comxim turntable that integrates with the shutter release of the camera. To standardize size and color, we place a ruler and an X-Rite Colorchecker below each specimen. The footprint of the entire physical set-up, including the camera and tripod, is 1 m x 1.5 m.

### Image capture

Before image capture, we record the specimen catalog number to a spreadsheet, and we take a photo of the color chart to assess ambient light later during processing. For each photograph, we use an f22 aperture for maximum depth of field, and we drop the ISO completely to avoid noise. For 360-degree image capture, the turntable is set to rotate 3.75 degrees between each photo, for a total of 96 photos taken over 7 minutes. This is repeated 3 times: one set of images is taken level with the specimen and a set is taken angled roughly 45 degrees above and below the specimen.

### Image processing

**Fig 2:**
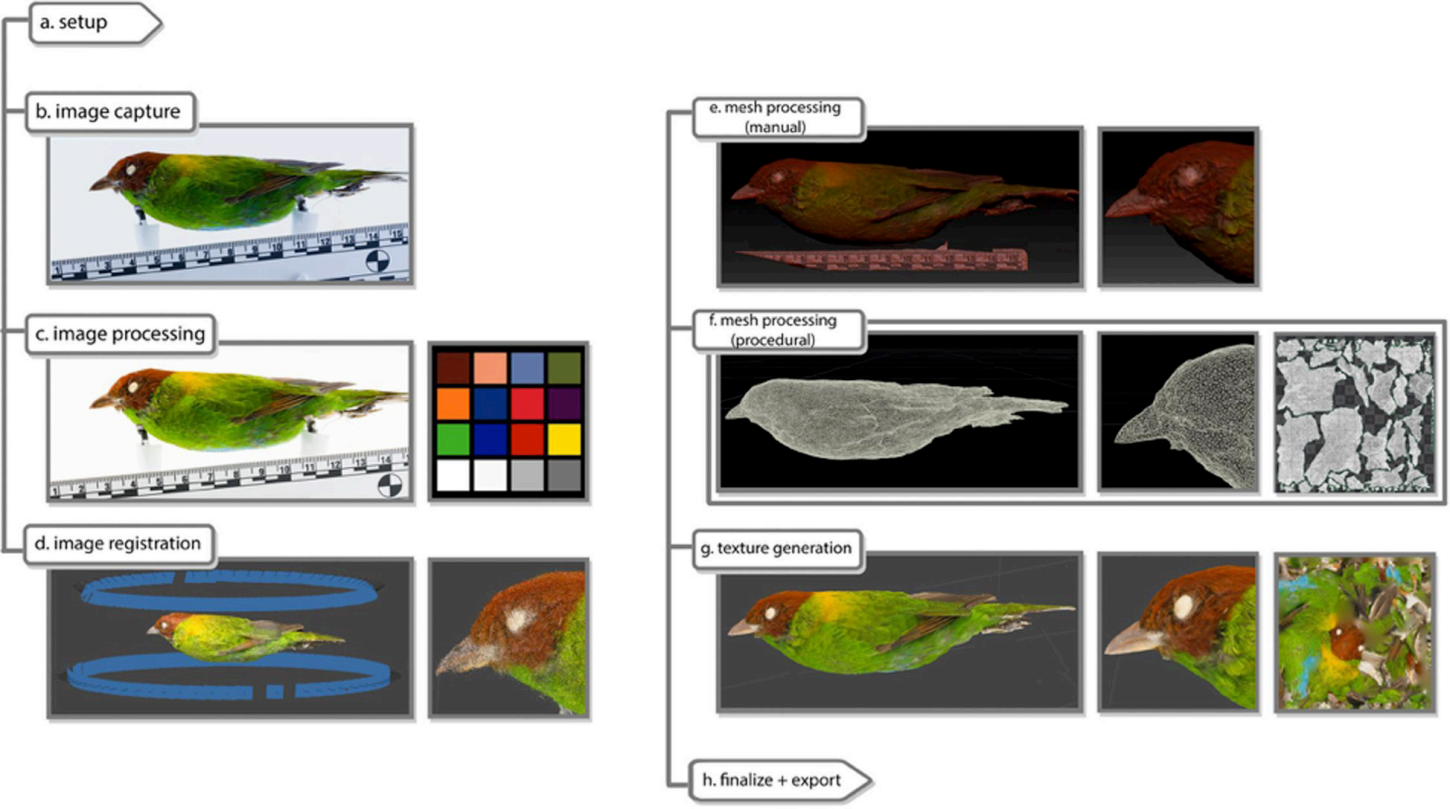
Overview of the workflow using a specimen of a rufous-winged tanager (*Tangara lavinia*), MLZ:Bird:8631. (a) Physical set-up shown in Fig 1; (b) One of 288 pre-processed RAW photos taken during the subject's rotation on the turntable; (c) Image processing using color chart to standardize lighting and color; (d) Image registration (alignment) where the 3D point cloud is surrounded by aligned camera locations, shown as blue rectangles (only two angles shown); (e) The 3D mesh generation from the point cloud includes extraneous detail, such as the scale bar, which can be removed after scale calibration; (f) Surface topology (shown in wireframe) optimized and UV coordinates (right) mapped onto the model; (g) Import into image registration software to add texture from the aligned photos, using UV coordinates (right).

The rest of the process to create a 3D model is carried out by a largely automated process (Fig 2) that requires minimal supervision (e.g., opening software programs, starting batch processing, etc.), except for a small amount of manual mesh processing (see below). For processing the scan data, we use a Windows PC with an intel i7 processor, 16 gigabytes of ram, and a Nvidia GeForce GTX1080 graphics card. The approximate time to completion of each step is listed in Table 2. The 288 photos (96 photos x 3 angles) are imported into Adobe Lightroom for image processing. Exposures and white balances are standardized using the 75% gray color chip, and photos are corrected for distortion and color aberrations using a color profile generated from the standard color chart. Photos are exported as high-quality JPGs. Then, within Adobe Photoshop, masks are created using a batch process that obscures the backdrop and isolates the subject in preparation for the next step: alignment of the 2D images to create a 3D model (image registration). The images are exported from Adobe Photoshop as JPGs with the “_masked” suffix.

**Table 2:** Average cost of each component of the pipeline.

Product	Cost in USD
camera (Sony A7rii)	$1,500
lens (Sony 90mm macro f2.8)	$1,000
lighting and turntable	$300
Adobe Photoshop and Adobe Lightroom	$10 monthly
Reality Capture (educational)	$40 monthly
SideFX Houdini	$200 yearly
Pixologic ZBrush	$40 monthly

### Image registration and mesh reconstruction

Images and corresponding masks are then loaded into Reality Capture and aligned, with the result visualized as a high-resolution point cloud with over 200,000 points. The alignment process takes approximately 10–20 minutes (Table 1). For collections use, Reality Capture provides speeds optimal for digitizing larger collections. Other software, such as Agisoft Metashape, might have advantages in other use cases that should be considered, especially for smaller collections [35]. The physical scale is then defined manually by placing markers on the standard ruler using 3–5 of the 288 photos. From the completed point cloud, a mesh is generated at a polycount of 3–6 million triangles and exported as an OBJ file.

### Mesh processing (manual)

While most models can be taken directly into procedural processing, we suggest using Pixologic Zbrush when manual quality control is desired. During manual quality control, the stand and other extraneous geometry attached to the specimen (e.g., specimen tag) are erased using ZBrush’s Sculptris tools. The export parameters are changed to export a triangulated mesh in the form of an OBJ file with a “_highpoly” suffix.

### Mesh processing (procedural)

**Fig 3:**
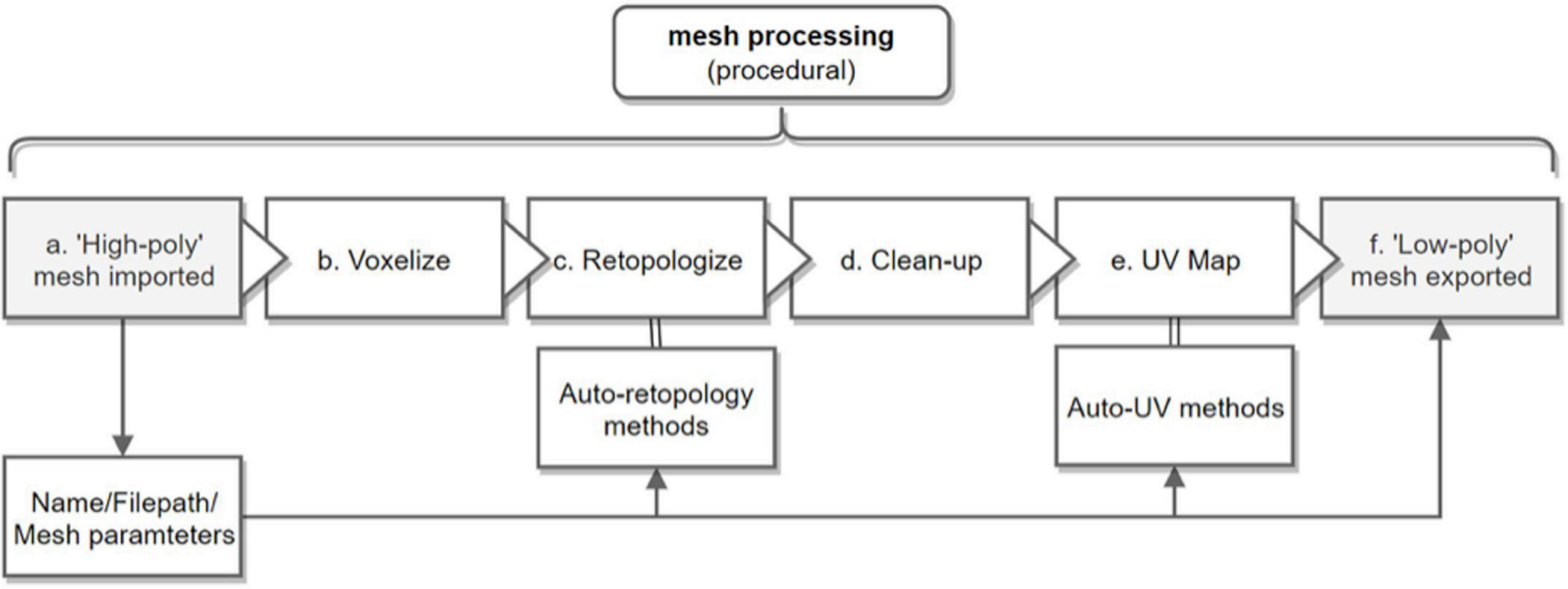
Detailed overview of the procedural mesh processing (Fig 2f), which follows a series of automated steps to process the 'high poly' 3D mesh into a 'low poly' optimized mesh. (a) Import: The mesh is imported, and its name and filepath is extracted for use during automation. Parameters affecting retopology and UV mapping can be modified during import; (b) Voxelize: To prepare for retopology, the mesh is filtered through a voxel grid that ensures uniformly-sized surface topology. This voxel-mesh is created at a higher resolution than the original, and projected onto the original surface to prevent distortion; (c) Retopology: The model's 'polycount,' or number of surface triangles, is reduced and optimized. This can either be according to predefined angle tolerance, or based on a predefined heatmap selecting areas of interest to be preserved at high-resolution; (d) Clean-up: Holes in the mesh, non-triangular and non-manifold geometry, and other topology errors are located and fixed; (e) UV map: UV coordinates can be created or "unwrapped" using a series of automatic projection methods, depending on the subject's shape. For most birds, we use 8 simultaneous planar projections placed according to the model's bounding box. This method uses Angle-Based flattening; (f) Export: The finished 'low-poly' mesh is exported and renamed according to the initial name and filepath.

After manual mesh processing, the model goes through a series of procedural mesh changes automated using SideFX's Houdini software (see Fig 3 and legend for details). First, the polycount is reduced through decimating the mesh according to a "quality tolerance,” which can be manually ‘painted on’ via a heat map to prioritize important features on the model’s surface [36]. Next, topology defects, such as holes and extraneous scan data are automatically recognized and removed. The mesh is aligned, and a further node can be toggled to retopologize the mesh into quads if so desired, with polygon edge flow dictated by a choice of presets, which can take cues from the “quality tolerance” heatmap previously applied. The mesh is then given UV coordinates generated from Houdini's automatic UV toolkit. In this context, UV does not refer to ultraviolet, but rather to coordinates of texture mapping (i.e., UV in addition to XYZ coordinates). We found Houdini’s automatic UV seam generation to be more efficient than those found in Metashape, or even those in Zbrush or Blender, and can be further tailored by reusing the heatmap initially used for retopology. The processed mesh is then exported as an OBJ file with a “_lowpoly” suffix. This file acts as the most optimized version of the model to texture and upload.

### Texture generation and final optimization

The “_lowpoly” model is then imported back into Reality Capture. From here, the aligned photos are used to generate either a 4k or 8k albedo texture map using the previously generated UV coordinates. This texture is exported into the same folder as the finalized “_lowpoly” OBJ file. The model is loaded into a 3D viewer for final inspection of the scale, texture, and placement in the 3D environment. Additional guidelines for file storage and optimization are found on the Smithsonian 3D metadata digitization blog (https://dpo.si.edu/blog/smithsonian-3d-metadata-model) and Morphosource’s data management guidelines [37].

### Ethics statement

The individual in Fig 1 has given written informed consent (as outlined in PLOS consent form) to publish this photo.

## Results and discussion

We produced a set of 40 3D models representing most of the type specimens in the Moore Laboratory of Zoology (MLZ) bird collection (S1 Table). These models are freely available for viewing and downloading at Sketchfab (https://skfb.ly/6PMr9 & https://skfb.ly/6PMru). A detailed guide for implementing the software pipeline—including code that automates file creation, image masking, and image storage—is available at [link here before publication]. The hardware set-up requires little physical space (1 x 1.5 m) and around $3,000 in initial investment, while the software pipeline requires $1,400/year for proprietary software subscriptions (Table 2). There are open-source alternatives (Table 3), although we have not incorporated them into our current pipeline. When using Reality Capture to process the scan data, the creation of each 3D model takes 1–2 hours/specimen and much of the software pipeline is automated with minimal supervision required.

**Table 3:** Open-source software alternatives for various steps in the pipeline.

Stage	Software
Image Processing (Masking)	Gimp^1^, OpenCV^2^
Image Processing (Post-processing)	Darktable^3^, OpenCV^2^
Alignment/Mesh Reconstruction	AliceVision^4^, CloudCompare^5^
Mesh Processing (Manual)	Blender^6^, Meshmixer^7^
Mesh Processing (Procedural)	Blender^6^, MeshLab^8^, UVLayout^9^

^1^gimp.org

^2^opencv.org

^3^ darktable.org

^4^ github.com/alicevision

^5^danielgm.net/cc

^6^blender.org

^7^ meshmixer.com

^8^ meshlab.net

^9^ uvlayout.com

A comparison of morphometrics from both physical and digital specimens shows that digital measurements from 3D models are comparable to hand-taken measurements. Over 20 specimens, the average difference between digital and hand-taken measurements of bill length was less than 1 mm (= 0.78 mm). The average bill length for these 20 specimens (mostly different species) was ~14 mm, meaning that the average error between hand-taken and digital measurements was about 5%, well within the range of what is considered a high repeatability for hand-taken measurements by the same observer (informally >90%).

While this demonstrates that 3D models can yield morphometric data, some measurements, like tail length, require landmarks inside the feathers (e.g., the insertion point of the tail feathers into the skin), which are impossible to determine from digital models or photographs. Even basic bill measurements can be challenging for some species because the nares are obscured by feathers. It is important therefore to recognize that digital models will never replace physical specimens as the primary source for biodiversity data. Apart from the difficulty of measuring certain traits, a more general reason for the primacy of the physical specimen is that the scientific uses of specimens continue to expand with continued technological development. 3D models might capture the external features of specimens with amazing resolution, opening doors of access and data collection, but they will entirely miss other important aspects of the Extended Specimen (sensu [5]): DNA, proteins, microstructures, hidden structures, parasites, internal anatomy, and much more.

Some aspects of external morphology are difficult to capture with 3D photogrammetry. Complex textures such as shaggy barbules or velvety feathers may result in fidelity loss and a tendency to clump or merge. During photo capture, the movement of any loose or delicate features may lead to interrupted alignment downstream. We found that contour feathers were well captured by the detailed texture maps, including more detail in the feather barbs than the human eye is capable of focusing on at close range. However, the modeling software often struggled to isolate thin, flat, or complex structures, such as the tail, protruding single feathers, and the toes. We discuss potential improvements to the pipeline below.

### Comparison to other existing 3D photogrammetry methods

A few studies have employed 3D photogrammetry for particular questions and taxa [30, 31, 38] and it has found increasing use in paleontology [39, 40], but as far as we know there is only one other attempt at a method for mass digitization of natural history specimens using 3D photogrammetry. Nguyen et al. [27] introduced custom-built photogrammetry hardware for the 3D imaging of insect specimens. Compared to the method they outline, which has likely been modified since then, our method does not use custom-built hardware, and therefore requires about half the initial investment cost (Table 2). The Smithsonian Institution Digitization Program Office offers a gallery of publicly available 3D artifact scans, and provides a benchmark standard for sorting 3D data in a mass-digitization context.

### Automation and the importance of procedural mesh processing

A key to 3D digitization’s practical use in large collections is the minimization of staff time. Automating the camera setup reduces staff time for the physical photography, but it is the procedural mesh processing in our software pipeline that may offer the biggest advance in automation. Mesh processing comes after point-cloud generation and includes removing extraneous detail, optimizing model topology, and avoiding data loss during decimation. In a comparative study of 3D scanning techniques, mesh processing averaged 40 minutes of staff time per scan [17]. Mesh processing is known to represent a serious bottleneck in the digitization pipeline and represents the majority of required 3D graphics experience and training [34, 35].

To address this problem, we implemented a procedural, node-based mesh-processing tool in SideFX Houdini, which not only cut down on staff time, but also on training time and required incoming expertise. Adopting Houdini’s tools reduced our staff time to 5–10 minutes per scan with minimal to no oversight. Since it follows a procedure of automated batch ‘nodes’, an understanding of the node-graph is all that is needed to access the pipeline. While 3D graphics experience is helpful when modifying the pipeline, it is not necessary to run the pipeline, or even to troubleshoot it. An automated procedure has other advantages. When using multiple software packages, the pipeline’s accessibility can be threatened by inconsistent updates, changes in licensing, and other logistical issues. Consolidating small processes into a larger, procedural program like Houdini improves compatibility and access to earlier builds of the pipeline.

Automated mesh processing techniques are still largely considered experimental, often relegated to being ‘add ons’ to more general-use software and can be cumbersome, especially when working between software packages. Bot and Irschick [34] point out that Agisoft’s automatic UVs often come out fragmented, without proper seams, and can be difficult to work with. We noted the same issue when using Reality Capture. They also found that uniform mesh triangulation without accounting for quality tolerance or edge alignment can also be problematic. We found similar problems with decimation when applied uniformly across a model, even in dedicated 3D packages such as Blender and Autodesk Maya. Similarly, Veneziano et al. [36] found that uniform decimation can lead to a “massive loss of information,” and that mesh processing should account for protecting areas of interest, especially those with articulated or thin surfaces, before decimation occurs.

We addressed some of these issues with procedural node-based mesh processing in our pipeline. For example, using SideFX Houdini, we could ‘paint on’ nodes prior to processing that protected thin or complex areas from decimation, while optimizing flat surfaces that did not need polygon-dense detail to be accurate. The resulting heatmap helped optimize UV generation and inform other steps in the procedure. Procedural modeling can also bridge software programs that would otherwise take time and training to navigate individually. An example from our pipeline includes combining Instant Meshes, a useful mesh-processing tool, with a custom Taubin smoothing surface operator written in OpenCL and implemented visually as a node in Houdini’s node-graph interface [36]. Procedural mesh-processing addresses a largely overlooked stage of the 3D digitization process, one that will only increase in importance as 3D models become more public-facing and shared via web platforms.

### Other future improvements to the pipeline

Another area of potential improvement is hardware efficiency. Future implementations could take advantage of photogrammetry’s unique modularity, compared to other methods, where different lenses and cameras can be swapped depending on the size of the specimen and its surface detail. For bird specimens, a qualitative assessment of bird diversity suggested that approximately 90% of living birds could be digitized with no or only minor modifications to the set-up outlined here. A three-camera setup would reduce the image capture time from 20 minutes to 7 minutes. For improving model quality, a stationary specimen with rotating cameras could potentially allow for higher fidelity scans through improved software processing and reduced noise in the photos, at the cost of increasing the physical footprint of the hardware set-up. Certain software features could be modified to improve quality or efficiency. For example, the quality setting can be increased when building a point cloud during alignment, but at the cost of processing time. We are working on further automating the pipeline with code that removes as much manual oversight as possible (see Github link in Methods for latest protocol updates). Finally, we are also working on a version using open-source alternatives to proprietary software (Table 3).

With a processing time of 1–2 hours per specimen and minimal manual oversight, we estimate that one worker could complete 4 specimens on one computer during an average workday, amounting to about 1,000 specimens digitized by one person on one computer in one year. Given that entire stages of the computational processing pipeline can be batched during idle periods, such as between workdays, these productivity estimates are highly conservative. The throughput rate can be effectively multiplied based on the number of simultaneous workflows [28], with more cameras and PCs. It has been shown that the integration of photogrammetry with other scanning methods can help cover the weaknesses of individual methods [23, 27]. Photogrammetry is recommended for its cost-effectiveness, ease of implementation, and modularity [41].

### Public access

Web-accessible, publicly viewable 3D collections are a primary goal of future digitization efforts [42, 43]. Existing 3D platforms like Sketchfab and the Smithsonian Institution Digitization Program demonstrate the advantages and challenges of hosting 3D models online. Web-hosted 3D models that are not only accurate but also web-accessible poses their own set of challenges, as 3D models must be optimized for space efficiency and proper rendering using public viewing tools. Due to its advantages in color-texture fidelity, photogrammetry has unique value here. But its advantages are only useful once staff time has been invested to process the model. File size, topology, UV coordinates, and texture maps all must be properly managed when web-hosting 3D models. This once again highlights the importance of procedural mesh processing we describe above.

### Future directions: Color analysis

Currently, large-scale color data acquisition from museum specimens employs either spectrophotometry [44], 2D images [45, 46], or scans of illustrations [47]. All of these methods have limitations. Spectrophotometry assesses color via point sampling, and therefore does not allow for reasonable whole-organism color analysis. Despite artists’ best efforts, illustrations cannot always accurately portray color and cannot capture hyperspectral properties like ultraviolet reflectance. And 2D image analysis, while probably the best available method, is still limited by the number of specimen rotations used (usually three: front, back, and side) and might introduce error in the flattening process [48].

**Fig 4:**
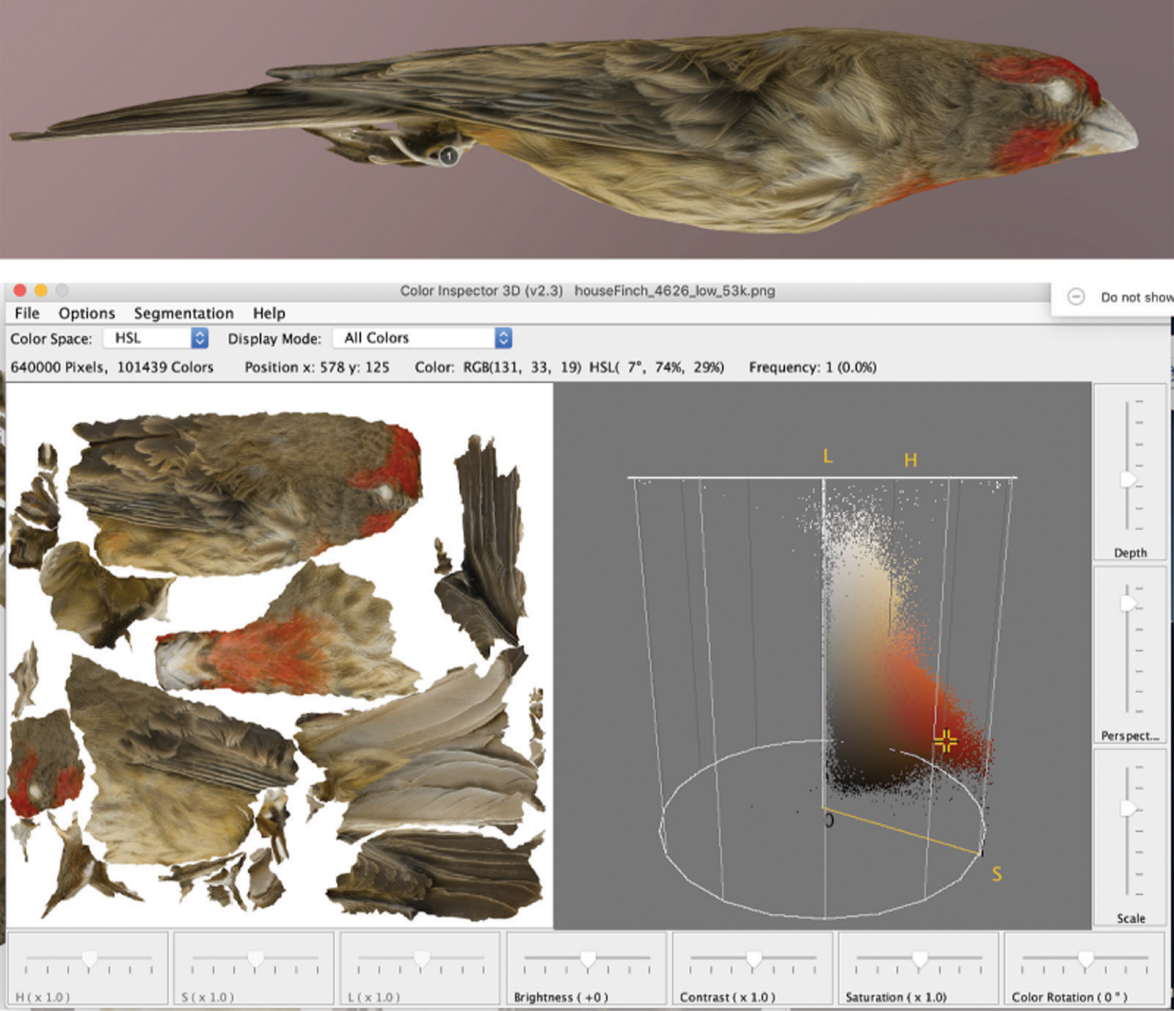
Sample color analysis of 3D models using a guadalupe house finch (*Haemorhous mexicanus amplus*) specimen, MLZ:Bird:65299. A 3D model (top) is broken into flattened components (bottom left) and each pixel visualized as a cloud of points in color space with ImageJ.

Color analysis is therefore one of the most promising immediate future directions for 3D photogrammetry, especially because methods like CT scanning, laser scanning, and structured light scanning are yet to be optimized for color capture. Though color options are available for laser and structured light scanning, they are limited by their hardware and do not match the resolution, sharpness, and detail provided by a mid-range camera sensor [40, 17, 41, 49]. While we did not specifically integrate color analysis into the pipeline, several open-source programs (like ImageJ; [50]) allow for detailed color analysis from flattened images created from 3D models (Fig 4). While most cameras filter out ultraviolet light—an important visual channel for many birds [51]—camera sensors can be modified to detect the ultraviolet range. Even certain reflectance properties like iridescence (i.e., changing color based on angle of light incidence) can be added during manual mesh processing and texture generation and visualized using real-time rendering engines, like those in Sketchfab and Morphosource [37].

This 3D photogrammetry pipeline is therefore a step toward a much-needed and more comprehensive method of color analysis based on continuous, whole-organism, full-spectrum color [52]. Combined with large-scale genomic data [53, 54] and complete phylogenies for various organismal groups [55, 56], color data from 3D digital models will help elucidate links between genotype and phenotype. Considering these links with other extractable phenomic data will open the door to new insights into their ecology, evolution, and functional morphology.

## Conclusions

3D photogrammetry is a promising method for capturing the external appearance of natural history specimens. It has been little used in natural history collections because no existing pipelines have proven efficient, cost-effective, and easy to set up. By introducing this pipeline for 3D photogrammetry, we hope to catalyze increased 3D digitization of the external features of specimens, which can complement 3D models of internal anatomy from CT scanning. The resulting phenomic data, collated across museums, will complement genomic data, opening new doors to the study of organismal ecology and evolution and the link between genotype and phenotype.

## Supporting information

S1 Table A list of the holotypes in each cloud and their relevant information. (XLSX)
